# Altered Erythropoiesis in Mouse Models of Type 3 Hemochromatosis 

**DOI:** 10.1155/2017/2408941

**Published:** 2017-05-02

**Authors:** R. M. Pellegrino, F. Riondato, L. Ferbo, M. Boero, A. Palmieri, L. Osella, P. Pollicino, B. Miniscalco, G. Saglio, A. Roetto

**Affiliations:** ^1^Department of Clinical and Biological Sciences, AOU San Luigi Gonzaga, University of Torino, Orbassano, Torino, Italy; ^2^Department of Veterinary Sciences, University of Torino, Grugliasco, Torino, Italy

## Abstract

Type 3 haemochromatosis (HFE3) is a rare genetic iron overload disease which ultimately lead to compromised organs functioning. HFE3 is caused by mutations in transferrin receptor 2 (TFR2) gene that codes for two main isoforms (Tfr2*α* and Tfr2*β*). Tfr2*α* is one of the hepatic regulators of iron inhibitor hepcidin. Tfr2*β* is an intracellular isoform of the protein involved in the regulation of iron levels in reticuloendothelial cells. It has been recently demonstrated that Tfr2 is also involved in erythropoiesis. This study aims to further investigate Tfr2 erythropoietic role by evaluating the erythropoiesis of two Tfr2 murine models wherein either one or both of Tfr2 isoforms have been selectively silenced (Tfr2 KI and Tfr2 KO). The evaluations were performed in bone marrow and spleen, in 14 days' and 10 weeks' old mice, to assess erythropoiesis in young versus adult animals. The lack of Tfr2*α* leads to macrocytosis with low reticulocyte number and increased hemoglobin values, together with an anticipation of adult BM erythropoiesis and an increased splenic erythropoiesis. On the other hand, lack of Tfr2*β* (Tfr2 KI mice) causes an increased and immature splenic erythropoiesis. Taken together, these data confirm the role of Tfr2*α* in modulation of erythropoiesis and of Tfr2*β* in favoring iron availability for erythropoiesis.

## 1. Introduction

Type 3 haemochromatosis (HFE3) is an autosomal recessive genetic disorder that leads to an accumulation of iron both in the blood and in several tissues, especially in the liver. Because of this, HFE3 patients have elevated transferrin saturation (TS) and serum ferritin level (sFt) [1].

This disease is caused by mutations in transferrin receptor 2 (TFR2) gene [2] that codes for two main isoforms, namely, Tfr2 alpha (Tfr2*α*) and Tfr2 beta (Tfr2*β*), that show moderate homology to the type 1 transferrin receptor (Tfr1) [3]. Unlike Tfr1, TFR2 gene expression itself is not directly regulated by iron [4] and TFR2 mRNA does not have iron responsive elements (IREs). Thus, there is no IRE-dependent posttranscriptional regulation of the protein levels [5]. Several in vitro studies have demonstrated that Tfr2*α* can bind iron loaded transferrin, however, with an affinity remarkably lower than that of Tfr1 [3]. Additionally, the levels of this protein in plasma membrane are regulated by TS, with an increased stabilization in the presence of highly saturated transferrin [6, 7]. The transcription of the entire TFR2 gene gives rise to the Tfr2*α* isoform which is a transmembrane protein. Tfr2*β*, on the other hand, is a shorter isoform lacking the cytoplasmic and transmembrane domains, and it is currently unknown if its activity is intracellular or extracellular. The expression patterns of these two isoforms are also very different. Tfr2*α* is highly transcribed in the liver and in erythroleukemic cell line (K562), while Tfr2*β* is mainly transcribed in liver, brain, heart [3], and splenic macrophages [8]. Most TFR2 mutations compromise the production of both Tfr2 isoforms. However, some mutations affect only the Tfr2*α* isoform, while others, such as M172K, abolish the Tfr2*β* methionine starting codon [9, 10]. 

Studies on murine models of HFE3 have demonstrated that Tfr2*α* and Tfr2*β* isoforms have distinct functions in iron homeostasis. While Tfr2*α* is involved in the hepatic pathway regulating iron hormone hepcidin (Hamp) [7], Tfr2*β* is involved in iron efflux from reticuloendothelial cells [8]. A recent study has demonstrated that the Tfr2*α* isoform is also expressed in erythroid progenitors. Here, it interacts with and stabilizes the erythropoietin receptor (EpoR), hence establishing a correlation between Tfr2*α* and erythropoiesis for the first time [11]. Furthermore, it is thought to be directly involved in the mechanisms of control of erythropoiesis, especially in conditions of iron deprivation [12, 13].

It is well known that erythropoiesis, as well as iron demand, changes throughout life in humans as a consequence of an increase in blood volume. Also, complete blood cell count (CBC) values differ slightly depending upon the age [14]. To our knowledge, no data exist to date on variations in erythropoiesis during ageing in mice. Some regulators of erythropoiesis have been characterized in recent years. One of them, named Erythroferrone (Erfe), is a hormone produced by erythroblasts that is able to regulate Hamp levels as a consequence of erythropoietic demand due to blood loss [15] or anemia of inflammation [16].

To investigate how Tfr2*α* silencing influences erythrocyte production across lifespan in mice, we studied erythropoiesis in two primary erythropoietic organs, the bone marrow (BM) and the spleen, at different ages using two mouse models with inactivated Tfr2*α* and/or Tfr2*β*. The findings of this study could be an important step toward gaining a better insight into Tfr2 involvement in erythropoiesis in humans.

Our results indicate that the lack of Tfr2*α* influences BM and splenic erythropoiesis starting from an early stage of life. Moreover, Tfr2*β* also influences erythropoiesis by the modulation of iron availability for erythrocyte maturation. More importantly, we now show that Erfe expression is regulated by erythropoiesis not only in adult animals, as previously demonstrated [15], but also in young age. Additionally, Erfe appears to be negatively modulated by erythropoietic tissues' iron availability. Lastly, we also describe the physiological variations of erythropoietic activity in WT mice during ageing.

## 2. Materials and Methods

### 2.1. Animals

Two Tfr2 mouse models on 129X1/svJ strain were studied: (1) Tfr2 KI that has the Tfr2*β* isoform inactivated (*α*^+^*β*^0^) and (2) Tfr2 KO that has both Tfr2 isoforms inactivated (*α*^0^*β*^0^). Selective targeting of Tfr2 isoforms was obtained starting from the same target construct in which murine M163K mutation (homologous to M172K human variant) was inserted in murine Tfr2 gene exon 4 flanked by 3 loxP sites activated through Cre/lox recombination system [8].

At adult age (10 w), Tfr2 KI mice have normal Hamp and normal serum iron parameters but splenic iron overload, while Tfr2 KO animals have normal Hamp but high serum ferritin and transferrin saturation as well as hepatic iron overload [8].

All animals were housed at Department of Veterinary Sciences, University of Torino. Animal housing and all the experimental procedures were performed in accordance with European (Official Journal of the European Union L276 del 20/10/2010, Vol. 53, p. 33–80) and National Legislation (Gazzetta Ufficiale n° 61 del 14/03/2014, p. 2–68) for the protection of animals used for scientific purposes.

Tfr2-targeted mice and controls were maintained on standard conditions and with ad libitum access to food and water. They were analyzed at 14 days and 10 weeks of age. Only male mice were used in this study to minimize potential variability related to sex.

At least 6 animals were analyzed for each experimental condition.

### 2.2. Hematological Analysis

Peripheral blood from the animals was subjected to complete blood cell count (CBC) analysis. Hemoglobin concentration (HB), hematocrit (HCT), erythrocytes number (RBC), and other indices (MCV, MCH, MCHC, and reticulocytes) were measured using an ADVIA®120 Hematology System (Siemens Diagnostics).

### 2.3. Flow Cytometry

Spleen and bone marrow (BM) were extracted from sacrificed animals and used for flow cytometric analysis using APC-Ter119 and PE-CD71. Fc-receptor was previously blocked using anti-mouse CD16/CD32; a mix of FITC-conjugated lymphoid and myeloid markers (CD3e, CD45R, CD41, CD11b, and Gr-1) was used to exclude leukocytes and 7-AAD was used to exclude dead cells. All reagents were purchased from eBiosciences. Ter119-positive events were allocated into five subsets representing sequential maturation stages (ProE, EryA, EryB, and EryC), according to CD71 intensity and FSC properties [17–19]. An EryD region was added according to the results by Chen et al., who localized a gate where orthochromic erythroblasts with nuclear-cytoplasmic dyssynchrony fall [19].

### 2.4. Histology and Perl's Staining

Animals livers and spleens were explanted, fixed in 4% PFA, cryoprotected by a sucrose gradient (7.5%, 15%, and 30%), and embedded in OCT prior to cryosectioning at 30 *μ*m. Tissue sections were stained with Perl's Prussian blue method (Bio-Optica). Images were taken at 20x magnification using a LEICA DFC208 microscope.

### 2.5. Monocytes/Macrophages Isolation

For each group (Tfr2 KI, Tfr2 KO, and control litter-mates), monocytes/macrophages were separated from a pool of 4 spleens using MACS CD11b MicroBeads (Milteni Biotec).

### 2.6. Molecular Analysis

Hamp gene expression was evaluated in Tfr2 targeted and in WT mouse liver. For reverse-transcription, 1 *μ*g of total RNA, 25 *μ*M random hexamers, and 100 U of reverse transcriptase (Applied Biosystems, USA) were used.

Hamp expression levels were measured by quantitative real-time reverse-transcription (RT-PCR) with CFX96 Real-Time System (BIO-RAD) using a quantitative RT-PCR assay (Assays-on-Demand; Applied Biosystems, USA). Erfe expression was evaluated using SYBR Green PCR technology (EVAGreen, BIO-RAD) using the following primers: mErfe F1 5′ATGGGGCTGGAGAACAGC3′ and mErfe R1 5′TGGCATTGTCCAAGAAGACA3′. All analyses were carried out in triplicate and results showing a discrepancy greater than 1 threshold cycle in 1 of the wells were excluded.* Gus* (*β*-glucuronidase) gene was used as housekeeping control. The results were analyzed using the ΔΔ threshold cycle (*C*_*t*_) method [20].

Western blots of BM, spleen, and monocytes/macrophages lysates (50 *μ*g) for Cleaved Caspase-3 (5A1E, Cell Signaling), Bcl-x_L_ (H-5), divalent metal transporter 1 (DMT1) (H-108), Fpn1 (G-16), Tfr2 (S-20), *β*-actin (C-4) (Santa Cruz Biotechnology), and Ft-L (kindly provided by S. Levi, Milan) proteins were performed through standard protocols.

Data from Western blot quantification (Image Lab Software, BIO-RAD) were obtained after normalizing on *β*-actin levels and expressed as fold increase, relative to the mean value obtained from the WT mice.

### 2.7. Statistical Analysis

For hematological analysis and flow cytometry experiments, statistical comparisons among genotypes and age groups were performed using nonparametric tests (Kruskal-Wallis or Mann–Whitney, resp.) using SPSS version 21 software. Differences of mRNA expression and protein production between controls and targeted mice were evaluated with a nonparametric Student's *t*-test (unpaired, two-tailed) using GraphPad Prism software. *P* < 0.05 was considered to be statistically significant.

## 3. Results

### 3.1. Peripheral Blood Cell Count

The pattern of erythropoiesis was observed to be different in WT and Tfr2 mice across the lifespan. Additionally, Tfr2 mice showed significant variations in erythropoiesis compared to age-matched WT controls in the CBC assay.

#### 3.1.1. Young versus Adult Animals

**Figure 1:**
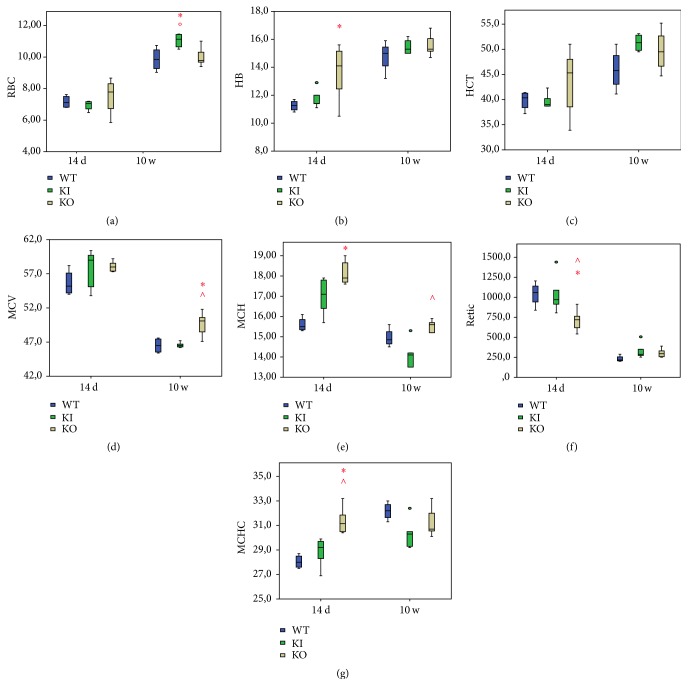
(a) Red blood cells (RBC; ×10E06 cells/*μ*L), (b) hemoglobin concentration (HB; g/dL), (c) hematocrit (HCT; %), (d) mean corpuscular volume (MCV; fL), (e) mean corpuscular hemoglobin (MCH; pg), (f) reticulocytes (retic; ×10E09 cells/L), and (g) mean corpuscular hemoglobin concentration (MCHC; g/dL) values obtained from animals CBC. WT: wild type (blue); KI: Tfr2 KI (green); KO: Tfr2 KO (yellow); 14 d: 14 days; 10 w: 10 weeks. *∗*  °  ∧ indicate statistically significant differences (*P* < 0.05) compared to age-matched WT, KO, and KI, respectively.

14 d old mice had lower RBC, HB, and HCT and higher MCV and reticulocytes compared to adults in all genotypes analyzed (Figure 1). Young WT mice showed similar MCH but lower MCHC as compared to adult WT mice, demonstrating that normal mouse erythropoiesis during animal growth follows the same trend as that of normal human erythropoiesis [14].

Young Tfr2 KI and KO mice demonstrated higher MCH but similar MCHC compared to adult animals with the same genotype (Figure 1). Thus, young Tfr2 mice have increased the levels of hemoglobin in RBC compared to age-matched WT animals. However, the differences are statistically significant only in Tfr2 KO mice.

#### 3.1.2. Young Tfr2 Mice

The comparison of 14 d old Tfr2 KI to WT mice did not reveal any significant differences in all CBC parameters analyzed. On the contrary, 14 d old Tfr2 KO mice had higher RBC, HB, HCT, MCH, and MCHC values than age-matched WT mice (significant differences for HB, MCH, and MCHC only) (Figures 1(b), 1(e), and 1(g)) and a significantly lower number of reticulocytes (Figure 1(f)).

To investigate if this difference in the number of reticulocytes in young Tfr2 KO animals could be due to an alteration of the apoptosis pathway in the BM, we analyzed Cleaved Caspase-3 (CC-3) [21] and Bcl-x_L_ [22]. The level of the proapoptotic marker CC-3 was comparable to that of age-matched WT mice, while the antiapoptotic marker Bcl-x_L_ increased about 1.5-fold in Tfr2 KO young animals versus WT (Figure S1 A, in Supplementary Material available online at https://doi.org/10.1155/2017/2408941). In conclusion, an increase in apoptotic death is not the underlying cause for the decreased reticulocyte production in these animals.

#### 3.1.3. Adult Tfr2 Mice

Adult Tfr2 KI animals had significantly higher number of RBC but lower MCH and MCHC compared to age-matched WT (Figures 1(a), 1(e), and 1(g)); however, these differences were not statistically significant.

Adult Tfr2 KO mice had statistically significantly increased MCV and MCH compared to WT litter-mates of the same age (Figures 1(d) and 1(e)). All the other CBC parameters were comparable to those of age-matched WT animals.

### 3.2. BM and Spleen Erythropoiesis

Flow cytometry analysis of the BM and spleen erythropoiesis revealed several unexpected results in both young and adult animals.

**Figure 2:**
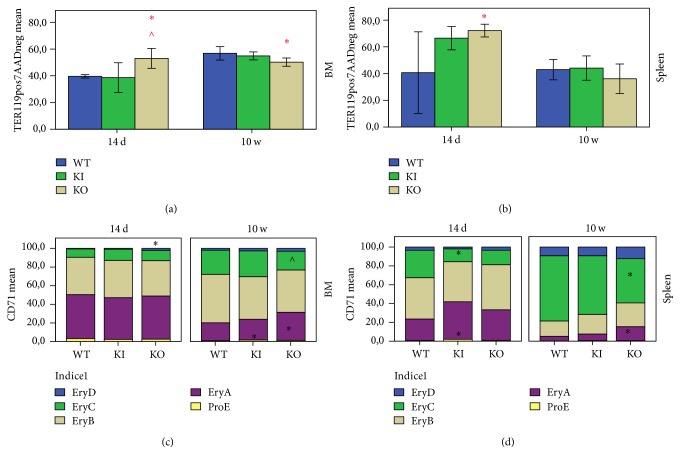
Flow cytometry analysis of quantitative (a, b) and qualitative (c, d) erythropoiesis in bone marrow (BM) and spleen of 14 days (14 d) and 10 weeks (10 w) old mice. WT: wild type; KI: Tfr2 KI; KO: Tfr2 KO. ProE, EryA, EryB, EryC, and EryD represent sequential erythropoietic maturation stages. *∗*  ∧ indicate statistically significant differences (*P* < 0.05) compared to age-matched WT and KI, respectively.

The BM erythropoiesis was increased in adult mice compared to the young ones (Figure 2(a)) in WT and KI mice alike. In contrast, Tfr2 KO mice had high levels of erythropoiesis already at 14 d of age, with values similar to adults (Figure 2(a)).

Both Tfr2 KI and Tfr2 KO 14 d old mice had increased splenic erythropoiesis compared to age-matched WT, though the difference was statistically significant only for the Tfr2 KO group. Interestingly, splenic erythropoiesis similarly reduced in adulthood for the two genotypes, reaching comparable values to the WT (Figure 2(b)).

From the qualitative point of view, no evident differences were found in the BM of young Tfr2 mice except for a higher number of EryD cells in Tfr2 KO (Figure 2(c)). Unexpectedly, an enhanced splenic erythropoiesis was observed in young Tfr2 KI mice, characterized by a left shift in the erythroid lineage resulting in a higher number of precursor erythroid cells and a lower number of mature cells (Figure 2(d)). Similarly, young Tfr2 KO mice also presented increased splenic erythropoiesis, as mentioned above (Figure 2(b)). However, no statistically different values were found compared to WT (Figure 2(d)).

Surprisingly, the erythropoiesis in adult Tfr2 KO mice was markedly altered. Although an increase in the percentage of early precursors (Figures 2(c) and 2(d)) was found, no reticulocytosis could be seen in these mice. This could be because of an increase in apoptosis as is evidenced by the doubling of the levels of the apoptotic marker Caspase-3 in adult Tfr2 KO mice compared to age-matched WT. On the contrary, the antiapoptotic marker Bcl-x_L_ was significantly decreased in Tfr2 KO mice compared to WT (Figure S1 B).

Finally, flow cytometry analysis revealed that in both young and adult Tfr2 KO mice CD71 MFI was significantly lower compared to WT in BM as well as in the spleen and in all erythroid maturation stages except for EryC in adult animals. CD71 MFI in Tfr2 KO mice was lower compared to KI mice for EryC in young animals and for EryA and EryB in adults (Figure S2).

### 3.3. Iron Levels in Tfr2 Animals

Since iron availability could influence erythropoietic stimulus, we analyzed the iron levels and hepatic Hamp production in erythropoietic tissues of Tfr2 animals as compared to controls.

**Figure 3:**
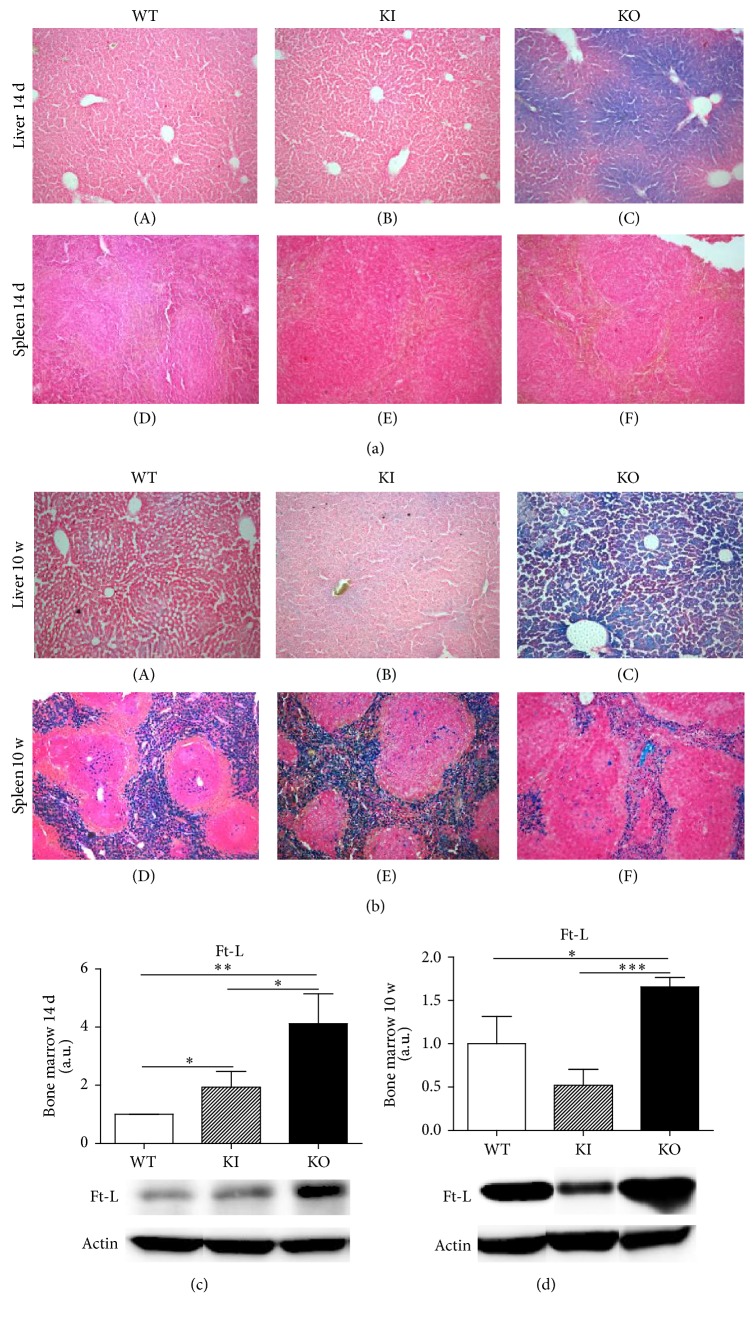
Perl's Prussian blue staining of liver and spleen sections from wild type (WT), Tfr2 KI (KI), and Tfr2 KO (KO) animals at (a) 14 days (14 d) and (b) 10 weeks (10 w) of age. Western blot analysis shows ferritin L (Ft-L) protein production in bone marrow of WT, KI, and KO mice at (c) 14 days (14 d) and (d) 10 weeks (10 w) of age. a.u.: arbitrary unit. The following symbols indicate statistically significant differences: ^*∗*^*P* < 0.05, ^*∗∗*^*P* < 0.01, and ^*∗∗∗*^*P* < 0.001 compared to age-matched WT mice.

Perl's histological staining (Figures 3(a) and 3(b)) revealed surprisingly high iron levels in livers from Tfr2 KO mice already at 14 d of age (Figure 3(a)) and, as expected, in adult animals as well (Figure 3(b)). In contrast, no significant liver iron deposit was visible in Tfr2 KI young and adult animals (Figures 3(a) and 3(b)). A significant splenic iron overload was evidenced only in Tfr2 KI adult animals (Figure 3(b)).

BM iron staining with Perl's did not reveal any obvious large iron deposit (data not shown). In addition, L ferritin (Ft-L) levels were found to be significantly elevated in the BM of both Tfr2 young mice (Figure 3(c)). However, during adulthood, it remained high only in the BM of Tfr2 KO mice (Figure 3(d)).

### 3.4. Hamp and Erfe Analysis

**Figure 4:**
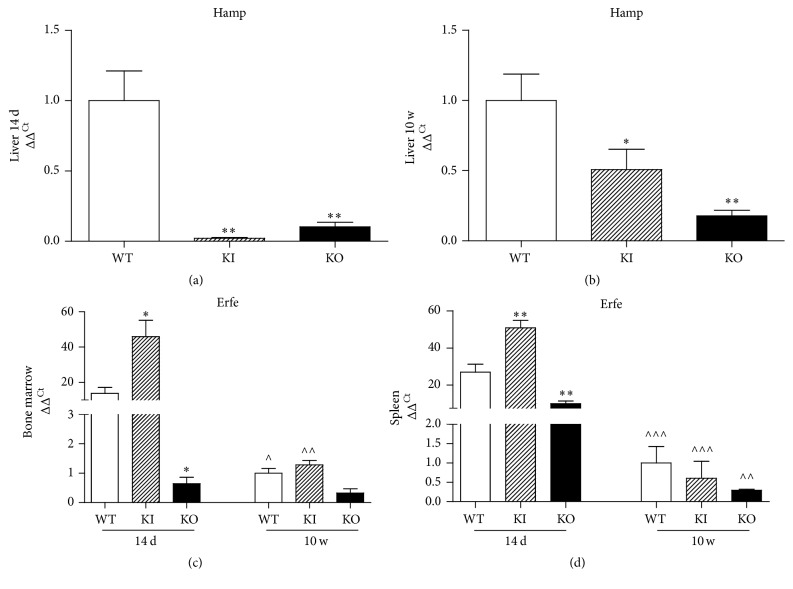
Hepcidin (Hamp) gene expression in Tfr2 targeted and in WT mouse liver at (a) 14 days (14 d) and (b) 10 weeks (10 w) of age. Erythroferrone (Erfe) gene expression in bone marrow (c) and spleen (d) of WT, KI, and KO mice at 14 days (14 d) and 10 weeks (10 w) of age. WT: wild type; KI: Tfr2 KI; KO: Tfr2 KO. The following symbols indicate statistically significant differences: ^*∗*^*P* < 0.05 and ^*∗∗*^*P* < 0.01 compared to age-matched WT mice; ^∧^*P* < 0.05, ^∧∧^*P* < 0.01, and ^∧∧∧^*P* < 0.001 compared to animals with the same genotype at 14 days of age.

The hepatic Hamp expression was significantly decreased in Tfr2 KI and KO animals compared to age-matched WT litter-mates. The same was true for adult animals, although to a lesser degree (Figures 4(a) and 4(b)).

Additionally, Erfe transcript levels were significantly different in the three genotypes: they were significantly higher in the BM and the spleen of young Tfr2 KI animals and significantly lower in young Tfr2 KO mice as compared to WT (Figures 4(c) and 4(d)). In adult Tfr2 KI and KO mice, Erfe transcription was similar to adult WT in both tissues analyzed (Figures 4(c) and 4(d)).

Longitudinal comparison between the two ages revealed that Erfe transcription was significantly increased in the young compared to genotype matched adult mice, with the exception of Tfr2 KO BM, whose Erfe amount remained constant during the growth period of the animal (Figures 4(c) and 4(d)).

### 3.5. Erythropoietic Tissues Monocytes

To unravel the role of Tfr2*β* isoform in the iron flux in macrophages during erythropoiesis, this cell type was isolated from the spleen of WT and Tfr2 targeted mice at the two experimental time points. Tfr2*β* levels were evaluated, together with the main proteins involved in cellular iron traffic, namely, the iron deposit protein ferritin (Ft-L), the iron importer DMT1, and the iron exporter Ferroportin 1 (Fpn1).

**Figure 5:**
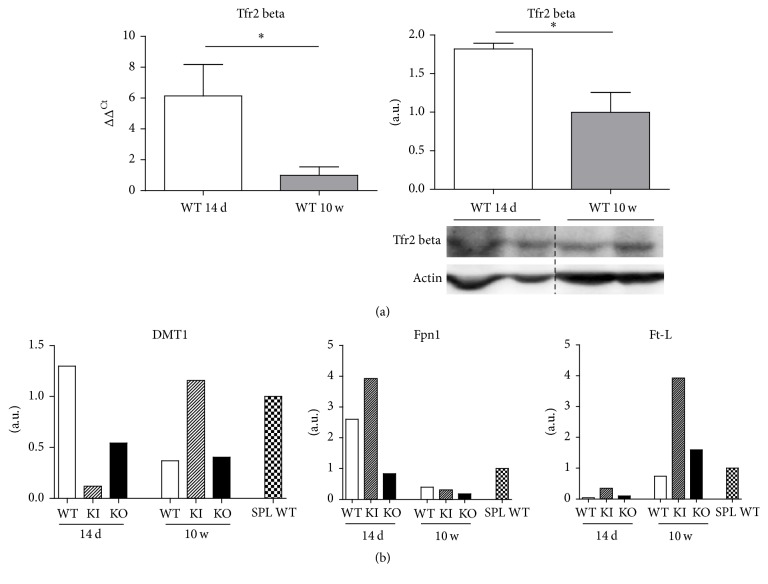
(a) Comparison between 14-day (14 d) and 10-week (10 w) Tfr2*β* expression (on the left) and production (on the right) in splenic macrophages of wild type (WT) animals. (b) Quantification of divalent metal transporter 1 (DMT1), Ferroportin 1 (Fpn1) and ferritin L (Ft-L) protein production resulting from Western blot analysis of splenic macrophages isolated from WT, KI, and KO mice at 14 d and 10 w of ages. a.u.: arbitrary unit; SPL: total spleen. *∗* indicates statistically significant difference (*P* < 0.05).

In WT mice, Tfr2*β* isoform was observed to decrease in splenic macrophages of adult animals as compared to the young ones (Figure 5(a)). In the same cells, DMT1 and Fpn1 decreased as well, while Ft-L levels increased (Figure 5(b)).

In young Tfr2 KI mice, splenic macrophages presented a lower DMT1 and higher Fpn1 and Ft-L compared to age-matched WT sib pairs (Figure 5(b)). During the growth period, DMT1 and Ft-L consistently increased while Fpn1 significantly decreased.

On the other hand, in young Tfr2 KO mice, splenic macrophages presented a lower DMT1 and Fpn1 and comparable Ft-L in comparison to age-matched WT sib pairs (Figure 5(b)). During the growth period, DMT1 and Fpn1 decreased while Ft-L was obviously increased.

## 4. Discussion

It is well known that iron is essential for adequate erythropoiesis. In the condition of iron deficiency, the most important pathway that is impaired is RBC production, firstly in the bone marrow (BM) followed by the spleen. Erythropoiesis itself undergoes physiological changes that reflect the requirements of an organism throughout its lifespan. It increases during youth, when there is massive body growth, but remains roughly constant during adult life and tends to decrease during ageing [23–25]. The adequate iron availability for this dynamic erythropoiesis is achieved through the modulation of hepcidin, one of the chief iron regulators [7].

Among the different hepcidin regulators, transferrin receptor 2 alpha (Tfr2*α*) has been shown to play a role as an iron sensor in the liver [7] and as erythropoiesis regulator in erythropoietic tissues [26]. Notably, the gene encoding TFR2 is transcribed in two main isoforms: the alpha form, expressed in the liver and few other tissues, and the shorter beta form, with a low ubiquitous expression. However, it is found to be in significantly higher levels in the spleen [3]. In the liver, Tfr2*α* exerts its action on the plasma membrane. It is not directly responsive to iron levels [4] but is stabilized on plasma membrane by iron loaded transferrin [27]. According to the most recent functional models, hepatic Tfr2*α* interacts with the other iron proteins as Tfr1 and Hfe, to sense body iron levels and to transduce the signal of iron excess through the activation of the Smad 1/5/8 and/or the Erk1/2 pathways, causing an increase in the hepatic hepcidin [7].

Recent data has demonstrated that Tfr2*α* also has an extrahepatic function. It is well expressed in BM, where it interacts with erythropoietin receptor (EpoR), thereby being involved in regulation of erythropoiesis [11]. Further, several studies have demonstrated the role of Tfr2*α* in regulating RBC production in mouse models, particularly in condition of iron deficiency [12, 13, 28].

In contrast, very little is known about the function of the second TFR2 isoform, Tfr2*β*. It is significantly produced in splenic macrophages and its silencing in the Tfr2 KI mice does not cause any variation in serum iron parameters and liver iron content. Nevertheless, these animals present iron retention in the macrophages probably through the downregulation of iron exporter Fpn1 [8].

Therefore, in the present manuscript, we aimed to analyze the role of both Tfr2 isoforms in erythropoiesis and the contribution of available iron in the modulation of erythropoiesis. We used the Tfr2 KI animals (*α*^+^*β*^0^), in which circulating iron levels are normal, and the Tfr2 KO mice (*α*^0^*β*^0^), that have severe iron overload in addition to increased serum ferritin and transferrin saturation. We compared the erythropoiesis in these animals to that of WT litter-mates. Furthermore, we evaluated these two Tfr2 mouse models at young age (14 d), when iron demand is high to fulfill growth requirements, and at adult age (10 w), when iron is needed primarily for the maintenance of erythropoiesis. Our findings demonstrate that adult Tfr2 KO mice show normal erythroid parameters at CBC except for an increased MCV and a higher hemoglobin content (MCH). This indicates that, in Tfr2 KO mice, the maximum amount of HB is produced in the RBC in the early stages of erythropoiesis, when cells are larger. On the contrary, in WT animals, the same hemoglobin amount reaches the final concentration through the reduction of RBC dimension. This phenomenon could be associated with an attempt of the body to eliminate the excess iron. In the same animals, BM and splenic erythroid production is quantitatively normal, but it is characterized by a shift toward immature precursors. The left shift in the maturation sequence could be an evidence of a delayed erythropoiesis in accordance with the results of previous studies [12, 13]. On the other hand, lack of reticulocytosis in these animals can be explained by an increase in the total BM apoptosis, confirmed by an increase of apoptotic marker Caspase-3 and a simultaneous decrease of the antiapoptotic protein Bcl-x_L_. This could represent a late stage control mechanism that may account for the depletion of late precursors that is ultimately responsible for an ineffective erythropoiesis.

Importantly, these findings are in contrast with the study by Nai et al., in which BM specific Tfr2 KO mice (Tfr2^BMKO^) present an increased number of RBCs, decreased volume and hemoglobin content, and increased splenic stress erythropoiesis, in the presence of normal serum iron parameters [28].

The primary genetic differences between these two models are that Tfr2^BMKO^ mice maintain Tfr2*α* and *β* isoforms function in the other body organs, particularly in the liver and in the spleen, whereas Tfr2 KO mice have a total silencing of both that causes an increased iron availability.

Therefore, the comparison between these two animal models is useful to unravel the role of iron in inducing RBC production in both the BM and the spleen. It is clear from the data that an increased iron availability in Tfr2 KO mice causes erythropoiesis, since 14 d old Tfr2 KO animals already have an erythropoietic activity similar to adult WT mice. In addition, it also causes erythropoietic changes, an increased number of immature cells, and increased apoptosis, as is evident in Tfr2 KO adult animals compared to age-matched WT. The increased iron levels in the BM of Tfr2 KO animals could trigger EryA erythroblasts production, but the increased apoptosis finally normalizes RBC output. Also, the presence of macrocytosis together with a low reticulocytes number and increased BM apoptosis in Tfr2 KO mice resembles the erythropoiesis of myelodysplastic syndromes (MDS) [29]. In these conditions, iron overload has been demonstrated to have a causative role. In fact, iron chelation of these MDS patients ameliorates their BM dysfunction [30].

It would be interesting to induce iron normalization in the Tfr2 KO animals to evaluate if a phenotype comparable to that of Nai et al. [28] can be achieved.

Another important difference between the two models is related to erythropoietic regulator Erythroferrone (Erfe) [15]. Erfe levels are increased in Tfr2^BMKO^ mice while decreased in our Tfr2 KO animals. Erfe increases due to an increased iron demand for erythropoiesis and causes a downregulation of hepcidin [28]. However, to our knowledge, this is the first time it has been demonstrated that Erfe reduction can also be a consequence of an adequate erythropoiesis, as we could observe in Tfr2 KO young animals.

Furthermore, the trend of Erfe in animals of different ages in both WT and Tfr2 targeted animals is very interesting. It clearly appears that Erfe has an important role in erythropoiesis regulation not only at adult age, as has been already demonstrated [16], but also at young age. Also, its expression correlates very well with erythropoietic boost in WT animals, being high in 14 d old animals and decreasing significantly in adult animals. Notably, BM and splenic Erfe transcription is significantly reduced in young Tfr2 KO animals and remains constant during their growth period, in agreement with the early achievement of adult erythropoiesis pattern in these mice.

The relationship between Erfe and Tfr2 has been demonstrated through several experimental approaches [13, 28]. Our data shows that this relationship is far more evident in young animals. Moreover, we demonstrate that the presence of Tfr2*α* in the liver is essential to have the hepcidin response to Erfe. In fact, Hamp is decreased in response to an increase of Erfe in Tfr2 KI mice, that produces the Tfr2 alpha isoform, as expected. Similarly, in Tfr2^BMKO^ animal model, a decreased Erfe amount corresponds to and increases Hamp level [28]. On the contrary, in Tfr2 KO animals, in which Tfr2*α* is absent in the liver, Hamp does not increase in response to low Erfe levels.

Lastly, the lack of Tfr2*β* leads to an evident splenic iron accumulation only in adult Tfr2 KI animals, as previously demonstrated [8]. Furthermore, it is surprising to see that lack of Tfr2*α* causes an iron accumulation as early as 14 d of age in the liver of Tfr2 KO animals.

We have focused at least a part of our analysis on young animals because very little is known about erythropoiesis at this stage of life even in WT animals. It is important to note that in the latter the erythropoietic stimulus is mainly iron-dependent, since an adult erythropoiesis becomes evident in iron enriched Tfr2 KO young animals.

Surprisingly, the data from the analysis of erythropoiesis in Tfr2 KI mice at young age is the most interesting, when these animals present normal serum iron parameters, normal BM and splenic iron amount, and normal CBC. In spite of this, their splenic erythropoiesis appears to be increased and immature compared to age-matched WT. These data are supported by a significant increase of BM and splenic Erfe as well. Tfr2 KO mice also present an early increased splenic erythropoiesis but it is qualitatively normal. This could be a consequence of the increased circulating iron availability that can ensure a qualitatively normal but relatively high splenic erythropoietic maturation.

Next, we hypothesized that enhanced splenic immature erythropoiesis in the absence of Tfr2*β* in young Tfr2 KI mice could be caused by low iron availability because of iron retention in splenic macrophages. This situation should have been evident in the spleen, where reticuloendotelial cells are particularly abundant. So, to confirm this hypothesis, we evaluated iron levels in splenic monocytes of Tfr2 mice. We detected an increase in ferritin and a decrease in Fpn1 in the splenic monocytes of young Tfr2 KI and KO compared to WT age-matched mice, which confirms our hypothesis.

Interesting speculations can be made from this analysis of the evolution of splenic erythropoiesis during the lifespan of animals.

From the analysis of WT animals, it is evident that the spleen eventually loses its role as erythropoietic organ and becomes a deposit site for iron, that derives from erythrocyte catabolism. Ft-L in fact increases in splenic monocytes of WT mice during ageing due to Fpn1 decrease.

The same is true in Tfr2 mice's splenic monocytes, but here the increase of iron is far more evident in splenic monocytes of adult Tfr2 KI mice compared to age-matched WT and Tfr2 KO. Also, iron importer DMT1 is increased in the splenic monocytes of Tfr2 KI mice. Thus, it could be interesting to further investigate the relationship between Tfr2*β* and this divalent metal transporter.

**Figure 6:**
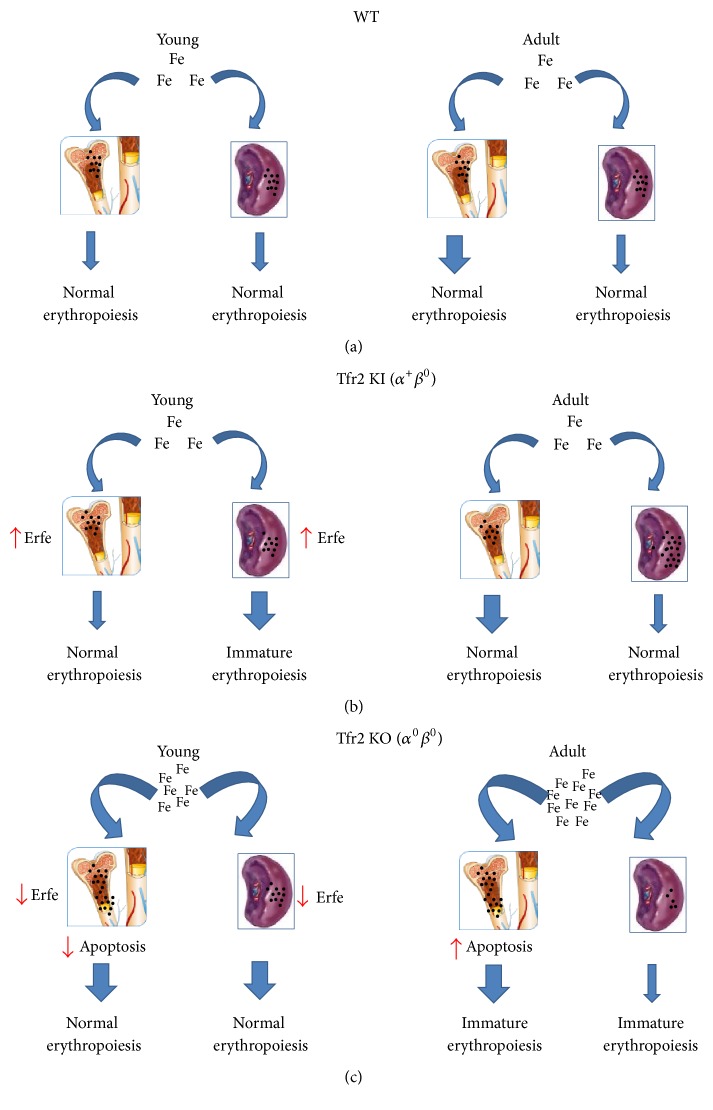
Model depicting the involvement of iron and Tfr2 isoforms in erythropoiesis. (a) In young (14 d) WT mice, erythropoiesis takes place in both bone marrow (BM) and spleen while in adult animals, BM erythropoiesis is predominant as compared to splenic RBC production (blue arrows); (b) in the presence of normal circulating iron levels, lack of Tfr2*β* provokes iron retention in macrophages that causes immature splenic erythropoiesis and Erythroferrone (Erfe) increase in young mice, that generally have a high iron requirement. This is normalized in adult animals, although an increased amount of iron is retained in the spleen; (c) the same should be observed in the spleen of Tfr2 KO animals, but they have sufficient circulating iron amount to normalize splenic erythropoiesis (Erfe is decreased). High iron availability also causes an increase of the early stage of BM erythropoiesis in adult mice, that does not result in reticulocytosis because of increased apoptosis.

On the basis of the data obtained in the present manuscript, a model for the role of iron in the stimulation of erythropoiesis at different ages and the involvement of Tfr2 isoforms in erythropoietic organs is illustrated in Figure 6.

## 5. Conclusions

An analysis of erythropoiesis in mice with inactivation of one or both of Tfr2 isoforms confirms that there is a specific function of Tfr2*α* in erythropoiesis, which has been previously demonstrated as well. Germinal lack of Tfr2*α* (Tfr2 KO) causes an anticipation of adult erythropoiesis in young mice in both BM and the spleen. On the other hand, lack of Trf2*β* is responsible for an increased but immature splenic erythropoiesis that is normalized during animal growth. This effect due to Trf2*β* absence in Tfr2 KO mice is compensated by the increased amount of circulating iron available for erythrocyte production.

## Supplementary Material

Supplemental material consists in two figures: Figure S1 reports the analysis of cellular apoptosis in Bone Marrow (BM). Figure S2 shows CD71 production decrease in Tfr2 KO and KI mice at specific maturation stages in BM and spleen at the two different ages.
