# Supplementary material for: Altered Erythropoiesis in Mouse Models of Type 3 Hemochromatosis
Source: Biomed Res Int. 2017 May 2;2017:2408941. doi: 10.1155/2017/2408941 (PMC5433419; doi:10.1155/2017/2408941)
Supplement: Supplementary file 1 — Supplemental material consists in two figures: Figure S1 reports the analysis of cellular apoptosis in Bone Marrow (BM). Figure S2 shows CD71 production decrease in Tfr2 KO and KI mice at specific maturation stages in BM and spleen at the two different ages. [file 2408941.f1.pdf]

ALTERED ERYTHROPOIESIS IN MOUSE MODELS OF TYPE 3 HEMOCHROMATOSIS (HFE3)

R.M. Pellegrino, F. Riondato, L. Ferbo, M. Boero, A. Palmieri, L. Osella, P. Pollicino, B. Miniscalco, G. Saglio, A. Roetto

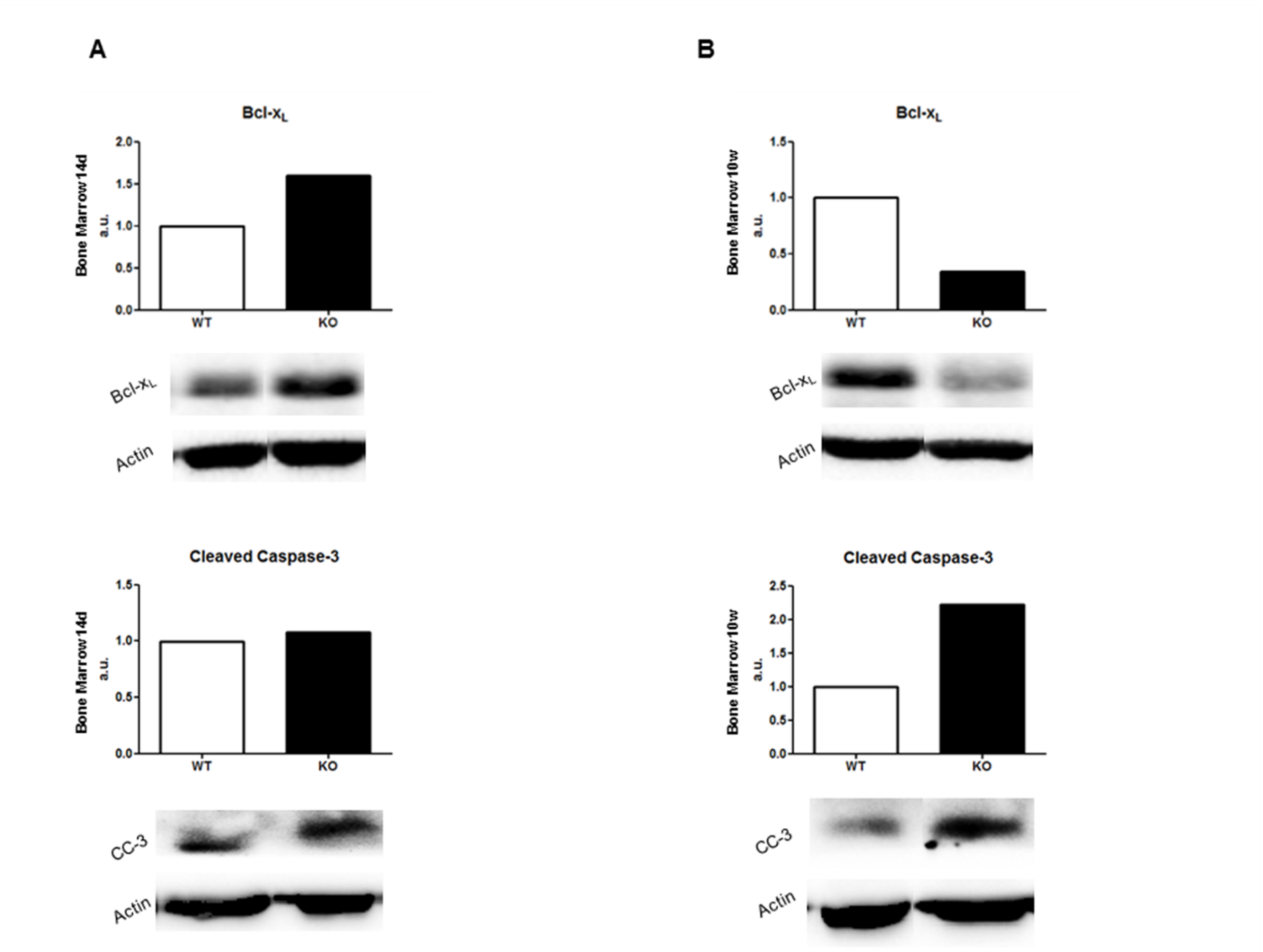

FIGURE S1

Western blot analysis show Bcl-x_L and Cleaved Caspase-3 (CC-3) proteins production in bone marrow of WT and Tfr2 KO mice at A)14 days and B)10 weeks of age. a.u.: arbitrary unit.

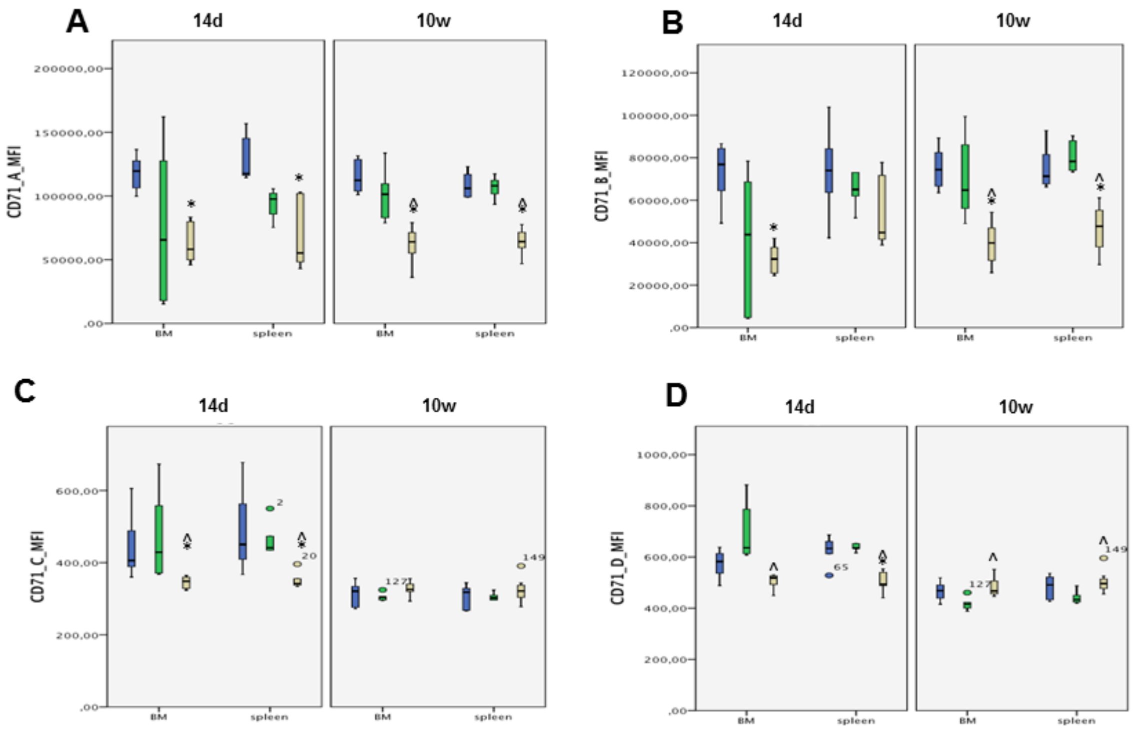

FIGURE S2

Flow cytometric analysis of mean fluorescence intensity (MFI) of CD71 in the different maturation stage of erithroyd precursors (A: EryA; B: EryB, C: EryC, D: EryD) in BM and spleen. blue: Wild Type; green: Tfr2 KI; yellow: KO. 14d: 14 days; 10w: 10 weeks
